# Post-processing of a distributed source method for the localization of somatosensory cortex in a cohort of epilepsy patients

**DOI:** 10.1016/j.ynirp.2024.100204

**Published:** 2024-05-09

**Authors:** Kevin Tyner, Matthew McCumber, Srijita Das, Carmen Urban, Anthony J. Maxin, Tiffany Chu, Mustaffa Alfatlawi, Stephen V. Gliske

**Affiliations:** a Department of Neurosurgery, University of Nebraska Medical Center, 988437 Nebraska Medical Center, Omaha, NE, 68198, United States; b College of Medicine, University of Nebraska Medical Center, 42nd & Emile Street, Omaha, NE, 68198, United States; c School of Medicine, Creighton University, 2500 California Plaza, Omaha, NE, 68178, United States

**Keywords:** Magnetoencephalography, Somatosensory evoked field, Singular value decomposition, Source localization

## Abstract

Localizing eloquent cortices is crucial for many neurosurgical applications, such as epilepsy and tumor resections. Clinicians may use non-invasive methods such as magnetoencephalography (MEG) to localize these cortical regions using equivalent current dipoles (ECDs). While dipoles are clinically validated, they provide the estimated strength, location, and orientation of only one or a few sources that best describe the recorded neuromagnetic data, requiring clinicians to make subjective decisions on the spatial extent of the underlying cortical area. More accurate delineation of eloquent cortical areas using distributed source localization methods would provide additional pre-surgical information on these regions’ location and spatial distribution, which could lead to reduced post-surgical complications associated with damage to or removal of eloquent cortices. Our objective in this paper was to present a method to post-process the distributed source localization results to yield a directly interpretable, distributed region of activation. As a test case, we selected somatosensory stimulation in a retrospective cohort of focal and multi-focal epilepsy patients. Our algorithm performs source localization using a distributed method (sLORETA), followed by post-processing and blind source separation to identify the area and boundary of the cortical tissue that primarily activates in response to somatosensory stimulation. We calculated the statistical significance of localization by comparing the identified region to an anatomical atlas and random chance. While examining patients who received left (upper left, UL) and right (upper right, UR) sided median nerve stimulation, the cortical areas identified by the algorithm were in anatomically appropriate areas with a median overlap of 97.6% and 94.7%, respectively. We observe that our algorithm localized somatosensory responses better than random chance in 57/58 (98%) patients who performed the UL task (*p* < 10 × 10^−10^, binomial test) and 49/50 (98%) patients who performed the UR task (*p* < 10 × 10^−10^, binomial test). We compared the localization of our algorithm to current clinical methods and found that our algorithm is not inferior to dipole localization. The algorithm can successfully localize somatosensory responses on the cortical surface in anatomically appropriate regions while providing the spatial extent of cortical activation, reducing subjectivity associated with dipole localization.

## 1 Introduction

Identifying eloquent cortices, brain regions related to fundamental neurological functions (Rosenow and Lüders, 2001), is crucial for patients who undergo surgical resection, such as those with epilepsy or tumors. Surgical resection risks damage or injury to adjacent eloquent cortices (Pondal-Sordo et al., 2006), and surgeons can prevent or minimize post-surgical deficits by avoiding resection of these eloquent areas. Therefore, defining the optimal plan for surgical resection depends on identifying tissue that must be resected and tissue that must be spared. As the exact location and spatial extent of eloquent cortices vary across individuals, having patient-specific information regarding the location and extent of eloquent cortices helps define the plan for surgical intervention and clarifies which subjects are candidates for surgical resection. One such clinical population that requires the identification of eloquent cortices are patients diagnosed with focal or multi-focal epilepsy.

Epilepsy, a brain disorder that leads to recurrent and unpredictable seizures (Zack and Kobau, 2017; Kitschen et al., 2023), is estimated to affect approximately three million people in the United States. Approximately 30% of those affected suffer from drug-resistant epilepsy (DRE) (Kalilani et al., 2018), experiencing recurrent seizures despite treatment with two or more anti-seizure medications (Ahrens et al., 2023). For these individuals, another treatment option is surgical resection, often preceded by intracranial electroencephalography (iEEG) monitoring over days to weeks in the hospital to plan the specific resection volume (Bernabei et al., 2023).

Functional mapping aims to pinpoint the precise cortical regions activated in response to task-related activities. The clinical gold standard for functional mapping is electrical stimulation mapping (ESM), which is an invasive method that involves applying low-level electrical currents through surgically implanted intracranial electrodes while simultaneously recording the resulting behavioral and electroencephalographic responses (Grande et al., 2020). However, it cannot be used to help inform the decisions needed before electrode placement, such as the ideal electrode location for stimulating specific eloquent areas. Therefore, ESM does not replace the need for non-invasive methods of localizing eloquent cortices.

Magnetoencephalography (MEG) is one non-invasive method used during pre-surgical localization of language (Lee et al., 2006), motor (Lin et al., 2006), and somatosensory (Ossenblok et al., 2003) cortices. Briefly, neuromagnetic responses elicited during a task-based MEG recording are averaged and source-localized to the patient's cortex via co-registration with the patient's MRI in a process known as magnetic source imaging (MSI) (Stefan, 2009). Solving an ill-posed inverse problem is required for source localization (Laohathai et al., 2021). While many source localization methods exist, discrete source modeling using equivalent current dipoles (ECDs) is the most prevalent (Tenney et al., 2020). Dipoles represent the location, strength, and orientation of one or a few cortical sources whose activity best describes the recorded neuromagnetic response (Tenney et al., 2020). Dipole analysis is used successfully in multiple contexts, including localization of the source of interictal epileptiform discharges in epilepsy subjects; see, e.g., Tamilia et al. (2019) and Rampp et al. (2019); interictal spike classification (Sdoukopoulou et al., 2021), and for the localization of P20/N20 sources (Antonakakis et al., 2019). While dipoles are the standard method available in FDA-approved software for MEG, they may not reflect the activity of the entire eloquent cortex volume. Additionally, before analysis, clinicians must determine the number of sources and channels used for localization (Tenney et al., 2020), and the method may fail if the underlying sources are not focal (Shiraishi et al., 2011). This method of localization introduces several subjective parameters into the analysis, which may reduce reproducibility and robustness (Nakasato et al., 1996).

In contrast to discrete source modeling methods, distributed methods utilize the entire brain as the source for the recorded neuromagnetic signal, preserving spatial information while not assuming the number of sources (Tenney et al., 2020). Such methods provide additional information on the spatial extent and temporal activation of cortical areas involved in task-based responses. Previous work has employed distributed source localization methods to identify motor and somatosensory cortices (Klamer et al., 2015), as well as language cortices (Raghavan et al., 2017). Distributed source localization methods have also been used to localize interictal epileptiform discharges (Pellegrino et al., 2018; Heers et al., 2016). However, as distributed methods produce maps of cortical activation that change in space and time (Tanaka and Stufflebeam, 2014), accurate localization of eloquent cortices can be difficult without additional post-processing.

In direct response to the limitations of distributed source localization, this work aims to assess and demonstrate the efficacy of a distributed source localization algorithm in identifying the cortical region that activates in response to somatosensory stimulation. We evaluated our algorithm using a retrospective cohort of focal and multi-focal epilepsy patients. Our algorithm first preprocesses the recorded MEG data and averages the somatosensory evoked responses. Evoked responses are then source-localized using standardized low-resolution brain electromagnetic tomography (sLORETA) (Pascual-Marqui et al., 2002). Here, we chose the sLORETA source localization approach as it has been shown to localize somatosensory responses consistent with ECD analysis (Terakawa et al., 2008) and has shown similar performance compared to MNE and dSPM for localizing somatosensory responses (Hedrich et al., 2017). Spatial filtering is applied to remove additional noise, and blind source separation using singular value decomposition (SVD) is performed to identify the dominant spatial and temporal modes. The spatial mode is then thresholded to identify the cortical regions that primarily activate in response to somatosensory stimulation. This paper builds upon our previous work by comparing our source localization approach to current clinical methods using a larger cohort of epilepsy patients (Tyner et al., 2023). Here, we chose to evaluate our algorithm using somatosensory responses as cortical regions involved in this response are reliably localized using ECDs with known spatial location and latency (Schaefer et al., 2004; Terakawa et al., 2008).

## 2 Materials and methods

### 2.1 Patients

**Fig. 1:**
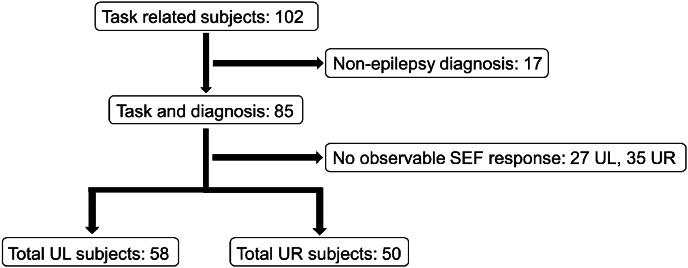
Consort diagram depicting patient inclusion/exclusion for the current study. From our retrospective database, 102 patients met the inclusion criteria. We excluded 17 patients for having non-epilepsy-related diagnoses. Of the remaining 85 patients, 27 patients who performed the upper left (UL) task and 35 who performed the upper right (UR) task were excluded due to no observable SEF response, resulting in 58 patients who performed the UL task and 50 patients who performed the UR task being included in the current study.

We selected patients who received MEG imaging as part of their clinical evaluation at the University of Nebraska Medical Center between September 2011 and September 2021. Our inclusion criteria were patients at least 19 years of age who had somatosensory mapping of the left (upper left, UL) and right (upper right, UR) median nerve during the recording, resulting in a cohort of 85 patients. Exclusion criteria included patients not diagnosed with focal or multi-focal epilepsy and patients lacking an observable response in the somatosensory evoked field (SEF) (27 patients for the UL task, 35 patients for the UR task), resulting in 58 UL patients, and 50 UR patients included in the current study. Of the patients included, 36 (58%) were diagnosed with focal epilepsy, 15 (24%) were diagnosed with multifocal epilepsy, and 11 (18%) had an unknown epilepsy diagnosis. See Fig. 1 for a consort diagram of inclusion and exclusion criteria and Table 1 for patient demographic information.

**Table 1:** Patient demographic information. Age is given as mean plus or minus standard deviation.

Feature	Total
Sex	36 F, 26 M
Age	35.4 ± 12.4
Handedness	53 R, 9 L
UL	58
UR	50

### 2.2 Somatosensory mapping paradigm

To localize somatosensory cortices, patients received electrical stimulation of either the left or right median nerve using an external cutaneous stimulator connected to a Digitimer stimulator system (Digitimer Ltd, Garden City, UK). Approximately 160–230 simulation events (mean ± std; 197 ± 31 for UL, 199 ± 31 for UR) were recorded for each patient with an inter-stimulus interval of 0.5 s. Each pulse had a duration of 0.2 ms and was a constant current square wave with the amplitude set to just above the patient's motor threshold (Burgess et al., 2011; Spooner et al., 2022).

### 2.3 MEG data acquisition

All MEG recordings were performed for clinical indications following standard of care clinical practice. The MEG data was recorded in a magnetically shielded room using a whole-head instrument equipped with 102 magnetometers and 204 planar gradiometers (MEGIN Neuromag, Helsinki, Finland). Before recording, the position of four head position indicator (HPI) coils, the bilateral pre-auricular and nasion points, and the overall head shape were digitized with a 3D digitizer (Fastrak 3SF0002, Polhemus Navigator Sciences, Colchester, VT, USA) (Spooner et al., 2022). MEG data were recorded with a bandwidth of 0.1–330 Hz and digitized at a sampling rate of 1 kHz. Noise reduction in the MEG data was performed using signal space separation with a temporal extension (tSSS) (Taulu and Hari, 2009). In three patients, additional electrodes were placed to measure electrocardiography (ECG) and electrooculography (EOG) signals.

### 2.4 MEG data preprocessing

We sought to calculate evoked responses resulting from somatosensory stimulation. MEG data were preprocessed in line with the previously established FLUX pipeline (Ferrante et al., 2022), using the MNE toolbox, implemented in Python (Gramfort et al., 2013, 2014). We first removed electrical interference by applying a notch filter at 60 Hz and its harmonics (60, 120, 180, 240, and 300 Hz; finite impulse response (FIR) filter; zero-phase; Hamming window; automatic filter length selection). For patients with ECG and EOG channels, we removed cardiac and ocular artifacts using signal space projection (SSP) (Uusitalo and Ilmoniemi, 1997). Additional artifacts, such as remaining ECG and EOG signals, channel noise, and stimulus interference, were removed by performing independent component analysis (ICA) using the Picard algorithm (Ablin et al., 2018). ICA has been shown to increase the signal-to-noise ratio of the data (Haumann et al., 2016) and is an acceptable preprocessing technique when only components that are associated with artifacts are removed (Ferrante et al., 2022). The data were then filtered from 2 to 120 Hz using an FIR bandpass filter with other parameters the same as the notch filter. We chose this frequency range to match the range used for clinical analysis of SEF responses at our institution. Epochs were created by extracting the data from 50 ms before to 250 ms after electrical stimulation, with the first 50 ms being used for baseline correction (Bowyer et al., 2020). Somatosensory evoked fields (SEFs) were generated by averaging all epochs (>100 epochs in all cases), and no epoch rejection was required.

### 2.5 Dipole analysis

To compare the accuracy of our algorithm against standard practice, we sought to localize SEFs as discrete activation sources using dipole analysis. We selected two latencies commonly used in dipole analysis: P20m and P40m (Hoshiyama et al., 1997; Kakigi, 1994), which occur 20 and 40 ms post-stimulus, respectively, in line with previous methods (Bowyer et al., 2020; Kakigi et al., 2000). We selected a third latency for analysis, henceforth referred to as PMaxm, which corresponds to the time point associated with the maximum root-mean-square (RMS) value of the gradiometers, measured between 15 and 60 ms post-stimulus, as magnetic responses in this range tend to localize to the primary somatosensory cortex (Kakigi, 1994; Kakigi et al., 2000). We chose this additional time point for analysis as there is biological variability in the time of maximum SEF response. Here, we calculated RMS as:

(1) $$RMS=\sqrt{\left(x_{1}^{2}+x_{2}^{2}+\cdots +x_{n_{\text{chan}}}^{2}\right)/n_{\text{chan}}}$$

where *x*_1_, *x*_2_, …, $x_{n_{\text{chan}}}$ represent the field gradient of each of the 204 gradiometers. For the P20m and P40m responses, single dipoles were fit at each instant from 20 ± 5 ms and 40 ± 5 ms post-stimulus. The dipole chosen from each time bin was the dipole with the highest goodness of fit (GOF). Note that we did not directly delay the time range for the P20m or P40m. Instead, the times are relative to the stimulation, and the (±5) ms search range for fitting allows for a delay in the neuromagnetic response. We denote the position of dipoles in surface RAS coordinates, where (0, 0, 0) mm represents the center of the MRI recording volume, +x is to the patient's right, +y is anterior, and +z is superior.

### 2.6 MEG source imaging

Next, we sought to determine the distribution of activated cortical sources in response to somatosensory stimulation in a patient-specific manner. To perform source localization at the level of individual patients, we preprocessed individual patients’ MRIs using the *recon-all* function implemented in FreeSurfer to generate the required cortical models (Dale et al., 1999; Fischl et al., 1999). We generated transformation files using the coregistration algorithm in MNE to determine the relationship between the MEG sensors and the head of an individual patient in MRI space. We created a single-layer boundary element model (BEM) with no down-sampling and a conductivity of 0.3 S per meter (S/m) for each patient, in line with previous work (Ossenblok et al., 2003). We built the source space using a dipole grid of 10,242 dipoles per hemisphere (*ico-5* spacing) for 20,484 total sources, with an average spacing of 3.1 mm between sources. We determined the current density distribution on the cortical surface using standardized low-resolution brain electromagnetic tomography (sLORETA), as previous studies have shown it has near-zero localization error (Pascual-Marqui et al., 2002). We associated the location of each of the 20,484 sources with a specific anatomical structure using the Destrieux atlas (Destrieux et al., 2010). We extracted the corresponding activation time course of each source location for the duration of the evoked response.

### 2.7 Identification of primary somatosensory cortex

**Fig. 2:**
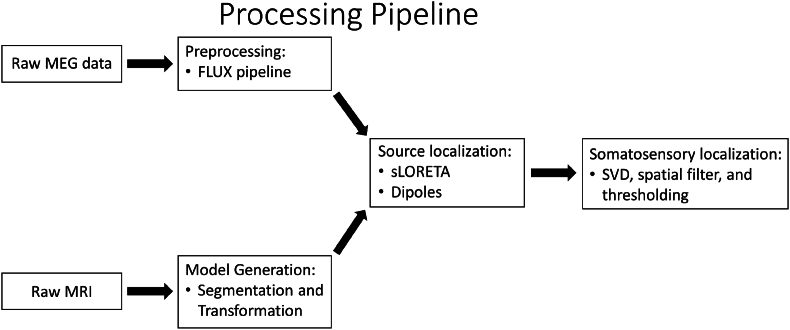
A diagram depicting the processing pipeline for MEG data. Raw MEG data is preprocessed following the previously established FLUX pipeline. Patients' raw MRIs are segmented and transformed using FreeSurfer. Preprocessed MEG data is then source localized using discrete (dipoles) and distributed (sLORETA) methods. Spatial filtering is performed, followed by blind source separation using SVD to identify the cortical area that is dominantly activated in response to the somatosensory task.

Our next objective was to delineate the boundary of cortical tissue primarily activated in response to somatosensory stimulation, in line with our previous work (Tyner et al., 2023). We first created models of the individual patient's pial surface using Fieldtrip in Matlab (version 2022a, MathWorks) (Oostenveld et al., 2011). We spatially filtered sources based on their anatomical location in source space with a search radius of 5 mm to remove additional noise. We then performed singular value decomposition (SVD), a blind source separation technique, on the individual patients' distributed source localized data from 15 to 60 ms post-stimulus. We defined the matrix **X** as the source localized data from *l* distributed source locations at each of *t* time points, representing the length of the analyzed window. Applying SVD to **X** yields:

(2) $$X=U\Sigma V^{\text{T}}$$

where the columns of the *l* × *t* matrix **U** are the left singular vectors, representing the spatial pattern of the activation sources or their distribution on the cortical surface, and the columns of the *t* × *t* matrix **V** are the right singular vectors, representing the temporal pattern of activation sources or its behavior over time. The diagonal non-zero entries of the *t* × *t* matrix **Σ** represent the variance explained by each activation source (Klema and Laub, 1980). To visualize the cortical area activated in response to somatosensory stimulation, we utilized the left-most dominant singular vector (first column of **U**) to represent the spatial distribution of the ionic current densities of the underlying dominant source. We selected this one component as it corresponds to the largest singular value and thus explains the most variance in the data. To identify the time at which the maximum response occurred, we identified the time of the maximum absolute value of the right-most dominant singular vector (the first column of **V**). See Fig. 2 for an overview of the data processing pipeline.

### 2.8 Overlap between discrete and distributed sources

We next sought to assess the consistency between the region our algorithm identified and the dipole analysis results. Since our algorithm results in a distributed source and the dipole analysis results in a single point, we decided to use two comparisons: 1) a distributed source comparison, converting the dipole point to a distributed source, and 2) an average distance comparison. In conducting these calculations, we rejected dipoles that were more than 9 mm away from pre- or post-central gyrus, as well as the central or post-central sulcus on the side contralateral to stimulation as dipoles for this task generally localize to these cortical regions (Kawamura et al., 1996; Kakigi, 1994; Niranjan et al., 2013).

#### 2.8.1 Distributed source comparison

We compared discrete and distributed sources by inflating the dipole point to a distributed source. We considered the source to be a sphere with a volume of 3 cm^3^ centered at either the P20m, P40m, or PMaxm dipoles. We chose this volume as it corresponds to the 95% confidence volume used for dipole localization of SEFs at our institution. We then calculated the percentage of distributed source points identified by the algorithm that overlapped with each sphere.

#### 2.8.2 Average distance comparison

To calculate the average distance, we first calculated the centroid of the distributed source. We then calculated the Euclidean distance between the P20m, P40m, and PMaxm dipoles and the centroid of the distributed source. We projected each point to the nearest source location on the cortical surface to identify the cortical regions associated with each dipole and distributed source. We recorded the associated anatomical location based on the Destrieux atlas.

### 2.9 Statistics

To assess the localization accuracy of our algorithm, we assessed the statistical significance in the following ways: 1) we compared the percentage of identified cortical sources that resided in the pre- and post-central gyrus, as well as the central and post-central sulcus, to random chance; 2) we compared the percentage of identified cortical sources that reside in a 3 cm^3^ sphere with the P20m, P40m, and PMaxm dipoles at the center, to random chance; and 3) we assessed the anatomical localization of the dipoles and the centroid of the distributed source. To compare to random chance, we randomly permuted, over the entire cortical surface, an area similar in size to the distributed source. We calculated the percent overlap with the expected anatomical regions and dipole volumes. This calculation was performed 10,000 times per task, and we evaluated statistical significance using a binomial distribution by identifying the number of patients where the median random overlap with the expected anatomical areas and dipole volumes was greater than the overlap with the distributed source, with *p*-values less than 0.05 being considered significant. To determine if there were differences in the anatomical localization, we projected the dipoles and the centroid of the distributed source to the nearest source location on the cortical surface and identified its anatomical label. We then identified the number of times each dipole and the distributed source were in the pre- or post-central gyrus, the central or post-central sulcus, or other brain regions. Statistical significance was assessed between the locations of each dipole and the distributed source using a chi-squared test, and *p*-values less than 0.05 were considered significant.

## 3 Results

### 3.1 Example of dipole source modeling

**Fig. 3:**
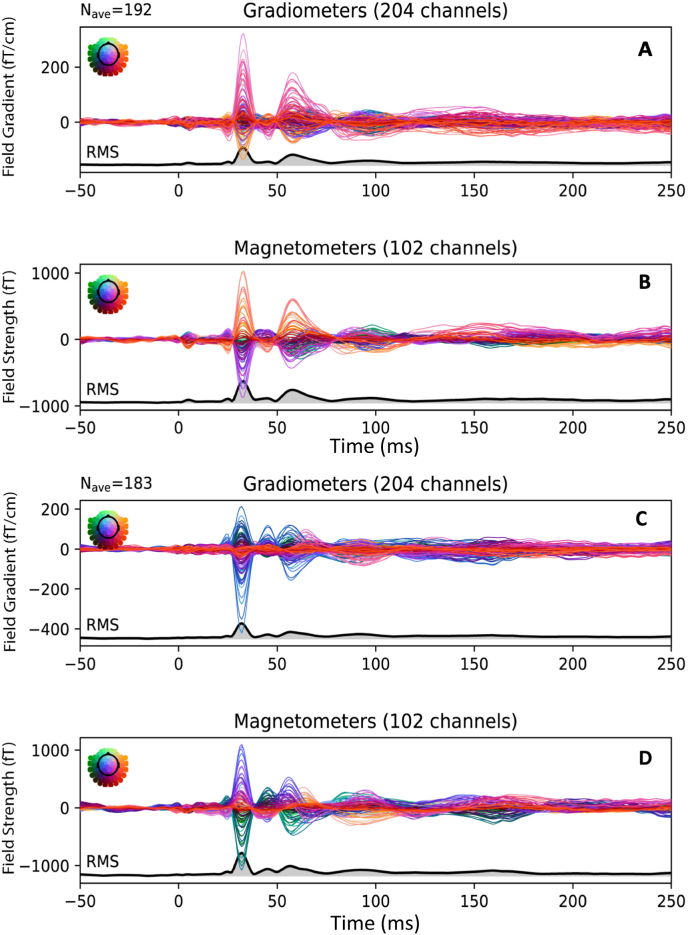
Evoked responses of a patient performing the upper left (A, B) and upper right (C, D) somatosensory task. Activity from each of the 204 gradiometers (A, C) and 102 magnetometers (B, D) are depicted, along with the root-mean-square (solid black line) of each channel type. The time ranges from 50 ms before to 250 ms after stimulation. The number of responses for each task is depicted by *N*_*ave*_.

**Fig. 4:**
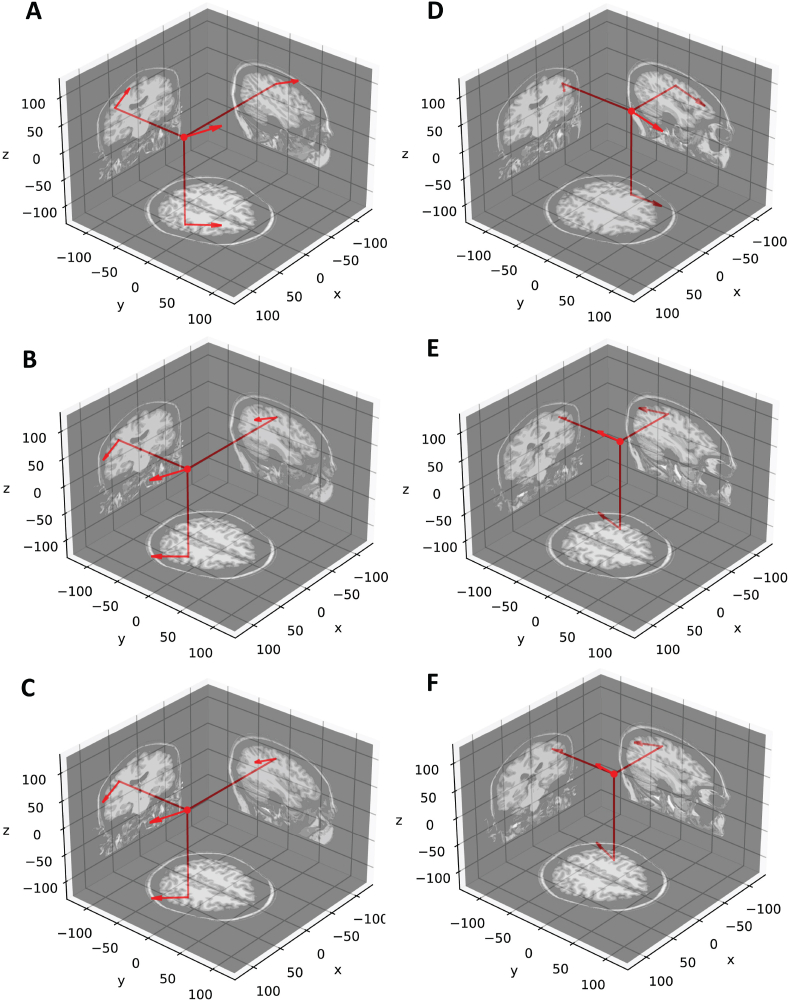
Dipole fits for a patient performing the UL (A–C) and UR (D–F) task. P20m dipoles are A & D, P40m dipoles are B & E, and PMaxm dipoles are C & F. For the UL task, the P20m dipole localized to the right post-central sulcus, the P40m dipole localized to the right central sulcus, and the PMaxm dipole localized to the right post-central sulcus. For the UR task, the P20m dipole localized to the left central sulcus, the P40m dipole localized to the right post-central sulcus, and the PMaxm dipole localized to the left post-central sulcus. Table 2 shows the relevant parameters for each dipole.

Using dipole modeling, we sought to localize the neuromagnetic recordings as discrete activation sources as an example of standard practice. We first preprocessed the patients’ data to generate an evoked response. An example evoked response is shown in Fig. 3. We then fit dipoles at approximately 20 and 40 ms post-stimulus, along with a third dipole, PMaxm, chosen at the time of maximum RMS in the evoked field, as measured by the gradiometers. Dipole localization for the same patient shown in Fig. 3 is shown in Fig. 4. Table 2 provides fit parameters for these dipoles.

**Table 2:** The time of fit, GOF, strength, and surface RAS coordinates for each dipole and task.

Dipole	Task	Time [ms]	GOF [%]	Moment [nAm]	x [mm]	y [mm]	z [mm]
P20m	UL	25	59.5	20.6	46.7	−16.5	35.3
P40m	UL	35	86.4	94.9	41.5	−17.0	36.0
PMaxm	UL	33	89.3	134.3	40.2	−17.9	36.3
P20m	UR	25	54.9	20.7	−41.4	−16.1	32.3
P40m	UR	35	66.1	53.4	−34.5	−29.4	39.2
PMaxm	UR	32	72.3	108.8	−34.6	−28.2	36.2

### 3.2 Example of distributed source modeling

**Fig. 5:**
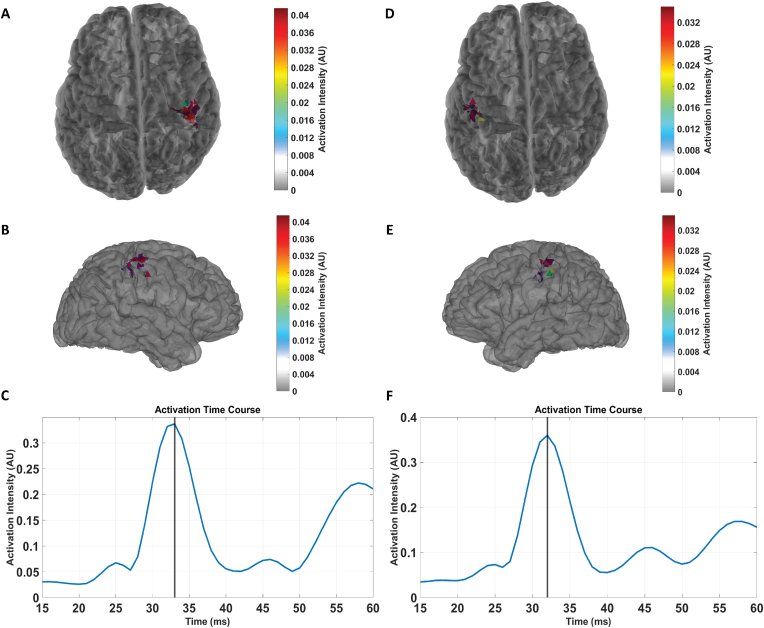
The activated cortical area and the temporal evolution of activation for the same example patient performing the UL (A–C) and UR (D–F) tasks. A, B, D, and E show the spatial distribution of the solution (A & D are coronal views, B & E are sagittal views). The spatial distribution is the left singular vector corresponding to the highest singular value, spatially filtered and thresholded to show the 80% strongest sources. The corresponding dipole fits are shown for reference: P20m (red triangles), P40m (yellow triangles), and PMaxm (green triangles). Most activity is localized to the pre- and post-central gyrus and the central sulcus in the hemisphere contralateral to stimulation. C & F) The dominant right singular vector represents the time course of activation. The solid black line depicts the time of maximum activation: UL (C) is 33 ms post-stimulus, and UR (F) is 32 ms post-stimulus. Dipoles for the UL and UR tasks localized to the contralateral central and post-central sulcus.

We next sought to model the underlying cortical activity of recorded neuromagnetic responses using a spatially distributed source model. Evoked responses were projected to the patients' cortical surface using sLORETA. We then performed spatial filtering on the patients' source localized data, followed by SVD and thresholding of the dominant left singular vector. This vector was then plotted on the subjects’ cortical surface to represent the activation of the underlying cortical area. The results for the same example patient are shown in Fig. 5, including both the spatial distribution and its time course.

### 3.3 Comparison with anatomical atlas

**Fig. 6:**
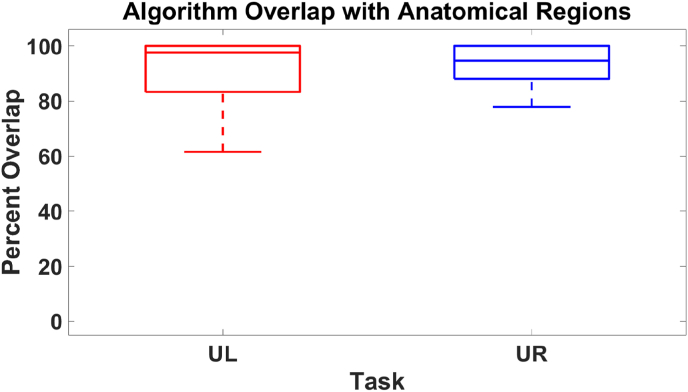
Box plot of the overlap of the percent of identified source locations that resided in anatomically appropriate regions. The median overlap for the UL task was 97.6%, while the overlap for the UR task was 94.7%. The algorithm did not favor localizing the UL or UR task.

We next sought to compare the results of our algorithm with anatomical regions using the Destrieux atlas. As dipoles localizing the primary somatosensory response for median nerve stimulation typically localize to the pre- and post-central gyrus, as well as the central and post-central sulcus contralateral to the side of stimulation, source locations identified by our algorithm that fell in these anatomical regions were considered anatomically correct (Kakigi et al., 2000; Tiihonen et al., 1989; Niranjan et al., 2013). However, as some patients may also have an ipsilateral response, source locations that resided in these regions ipsilateral to stimulation were also considered anatomically correct (Kanno et al., 2003). When comparing the algorithm against the atlas, we find that, in the UL task, a median of 97.6% of points resided in an anatomically appropriate area, while in the UR task, a median of 94.7% of points were anatomically correct across all patients, with no significant differences between tasks (*p* = 0.96, Wilcoxon signed rank); see Fig. 6.

We conducted a numerical simulation to assess whether these results differ from the random chance agreement. We found that a median of 9.0% of randomly generated points were anatomically correct for both tasks. We then identified patients where the algorithm was more accurate than random chance. We find that the algorithm is more accurate in 57/58 (98%, *p* = 3.5 × 10^−76^, binomial distribution) and 49/50 patients (98%, *p* = 8.8 × 10^−66^, binomial distribution) performing the UL and UR tasks, respectively.

### 3.4 Distributed source comparison

**Fig. 7:**
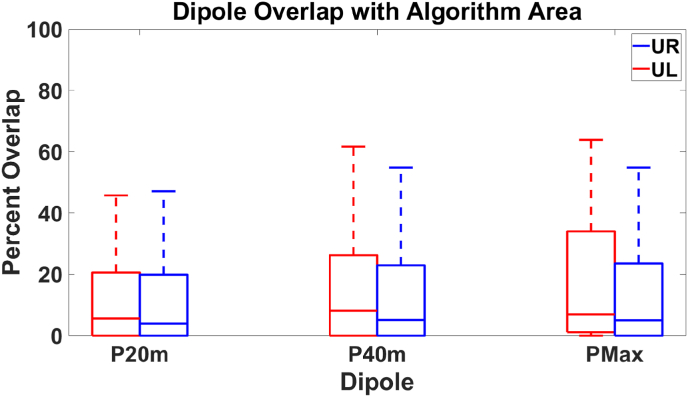
Overlap between the cortical area identified by the algorithm and a 3 cm^3^ sphere centered at the P20m, P40m, and PMaxm dipoles. For the UL task (red), there is a 5.6%, 8.2%, and 6.9% overlap with their respective dipole volumes, with corresponding *p*-values of 2.3 × 10^−26^, 2.0 × 10^−36^, and 6.1 × 10^−40^ (two-tailed Wilcoxon signed rank) when compared to the overlap of a randomly generated cortical area. For the UR task (blue), there is a median overlap of 4.0%, 5.1%, and 5.0%, with corresponding *p*-values of 1.1 × 10^−27^, 3.5 × 10^−27^, and 1.3 × 10^−30^ (two-tailed Wilcoxon signed rank) for the P20m, P40m, and PMaxm dipoles respectively, when compared to the overlap of a randomly generated cortical area.

We next examined the overlap between the sources identified by the algorithm and a 3 cm^3^ sphere centered at P20m, P40m, or PMaxm dipoles. Source locations identified by the algorithm that resided in the sphere were considered the overlap points. See Fig. 7. For the UL task, we find a median overlap of 5.6%, 8.2%, and 6.9% for the P20m, P40m, and PMaxm dipoles, with corresponding *p*-values of 2.3 × 10^−26^, 2.0 × 10^−36^, and 6.1 × 10^−40^ when compared to the overlap of a randomly generated area. For the UR task, we find median overlaps of 4.0%, 5.1%, and 5.0% for the P20m, P40m, and PMaxm dipoles, respectively, with *p*-values of 1.1 × 10^−27^, 3.5 × 10^−27^, and 1.3 × 10^−30^ (two-tailed Wilcoxon signed rank) when compared to the overlap of a randomly generated area.

### 3.5 Average distance comparison

**Fig. 8:**
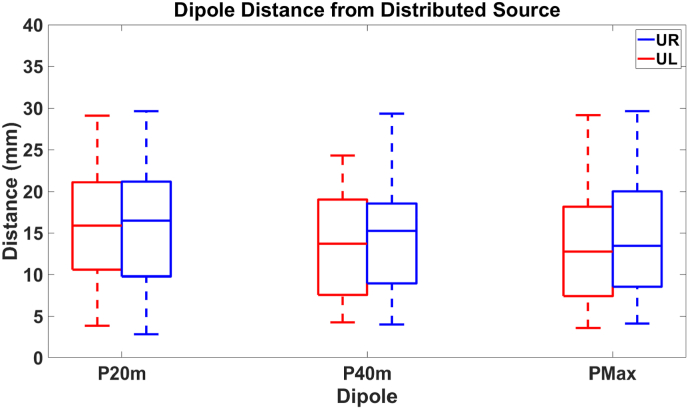
Distance between each dipole and the centroid of the distributed source. For the UL task, the median distances were 15.9 mm, 13.7 mm, and 12.7 mm for the P20m, P40m, and PMaxm dipoles, while for the UR task, the median distances were 16.5 mm, 15.2 mm, and 13.4 mm for the same dipoles, respectively.

Our next objective was to determine the average distance between the fit dipoles and the distributed source. For the UL task, the median distances were 15.9 mm, 13.7 mm, and 12.7 mm for the P20m, P40m, and PMaxm dipoles. For the UR task, the median distances were 16.5 mm, 15.2 mm, and 13.4 mm, respectively; see Fig. 8.

**Fig. 9:**
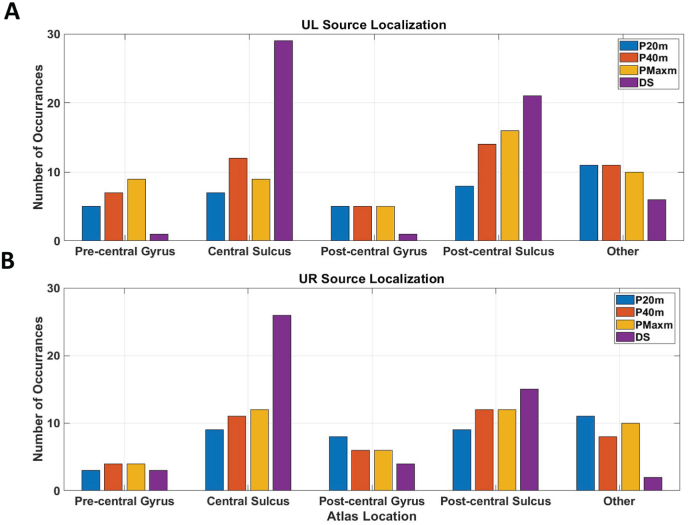
Atlas location for the P20m, P40m, PMaxm dipoles, and centroid for the Distributed Source (DS) for the UL (A) and UR (B) tasks. The number of times each dipole and centroid was located in the pre-central gyrus, central sulcus, post-central gyrus, post-central sulcus, and other brain regions are indicated. There are no significant differences in anatomical localization between each dipole and the distributed source (all *p* > 0.1, *χ*^2^ test).

We next sought to determine if there were differences in anatomical localization between the dipoles and the distributed source. We projected dipoles to the nearest source location on the cortical surface and identified their anatomical location based on the Destrieux atlas. See Fig. 9 for the anatomical location of each dipole and distributed source for the UL and UR tasks. We compared the number of P20m, P40m, and PMaxm dipole localizations in the pre- and post-central gyrus, central and post-central sulcus, and other brain regions to the anatomical localization of the distributed source. We find no differences in localization between methods (all *p* > 0.1, *χ*^2^ test).

## 4 Discussion and conclusion

This work aimed to demonstrate the efficacy of our distributed source localization algorithm to localize the primary somatosensory cortex from MEG recordings in a retrospective cohort of epilepsy patients. Our algorithm successfully localizes the somatosensory response in an expected anatomical region consistent with current clinical methods. Additionally, our algorithm provides a boundary of cortical activation instead of a single, discrete activation source provided by ECD modeling.

Our results indicate that the cortical area identified by the algorithm has a statistically significant overlap with the pre- and post-central gyrus and the central and post-central sulcus in nearly all patients, as seen in Fig. 6. To determine the anatomical accuracy of our algorithm, we defined identified sources that resided in expected anatomical regions in both hemispheres to be anatomically correct, as dipole fits for this task have been localized to these regions (Kakigi et al., 2000; Kanno et al., 2003; Tiihonen et al., 1989; Niranjan et al., 2013). We anticipate results further improving by the use of using more advanced inverse methods or head models, e.g. (Antonakakis et al., 2019; Rezaei et al., 2020).

We observed considerable variability in the overlap percentages, as seen in Fig. 6. This variability could be the result of several factors. First, although we visually rejected data with poor quality, some residual variability in data quality could be present. Our algorithm contains no explicit rejection parameters for data that may be of lower quality. Second, the current iteration of the algorithm only utilizes the first left and right singular vectors. As the first left singular vector may contain noise corresponding to background cortical activity or signal unrelated to the somatosensory response, it may be advantageous to examine the localization of other components. Third, as all patients received a clinical diagnosis of focal or multi-focal epilepsy, they may have atypical somatosensory representation. To the best of our knowledge, there has been no large-scale investigation into the impact of epilepsy on somatosensory pathways, but this possibility cannot be ignored as cortical reorganization of other networks, such as the language network, has been seen in this clinical population (Datta et al., 2013; Jensen et al., 2011). As reorganization of cortical networks may occur in epilepsy patients, there may also be variability in the temporal activation of cortical sources. The window used for analysis (15–60 ms post-stimulus) may not contain a prominent somatosensory response in all patients, reducing overlap with the expected anatomical regions.

We sought to determine if our algorithm would localize somatosensory responses in a cortical region consistent with current clinical methods by calculating the overlap between the cortical region identified by the algorithm and a point source inflated to a volume of 3 cm^3^ centered at P20m, P40m, and PMaxm dipoles. We chose this method as it allows us to determine if discrete and distributed sources are localized to similar cortical areas. We found that the percentage of our distributed source result that overlapped with the dipoles was relatively small, demonstrating that our results identified a significant amount of tissue not immediately adjacent to the dipole result. While we anticipate that this difference reflects a more accurate representation of the region of activated cortex, a few other factors could also impact this result. We used a relatively unbiased dipole localization method to localize the evoked response, apart from excluding dipoles located more than 9 mm away from the expected anatomical regions. Here, we utilized all MEG channels for localizing somatosensory responses. While dipoles primarily localize to the side contralateral to stimulation, some patients may have an ipsilateral response (Kanno et al., 2003). Clinicians typically focus on just the contralateral side. The unbiased nature of our dipole fits may alter dipole localization, resulting in a reduced overlap with the distributed source.

Given the relatively small overlap between the distributed source and the dipole results, we also wanted to assess the distance between discrete and distributed sources. As shown in Fig. 8, we found the median distance ranged from 12.7 mm to 16.5 mm depending on the dipole and task. This data suggests that the distributed source does not just extend beyond the region adjacent to the dipole but is also centered a bit differently than the dipoles. Given the available data, we cannot know which solution more accurately describes the eloquent cortex. To better understand this difference, we next sought to determine if there were differences in anatomical regions associated with the dipole and the distributed source results. We found no statistically meaningful differences in the anatomical localization of discrete and distributed sources (all *p* > 0.1) for the UL and UR tasks, although the general trend is that the distributed source is more consistently located in the central sulcus while the dipoles are more broadly spread over the two gyri and sulci.

The distance between dipoles and the distributed source may seem significant. However, previous work has shown a localization error of approximately 10 mm between the N20m dipole and the minimum norm estimate distributed source (Molins et al., 2008), while another reported a distance of 5 mm between the N20m dipole and a quantifiable sLORETA distributed source (Terakawa et al., 2008). While our distances were larger than previous reports, we attribute this difference to the localization method utilized, the number of patients included in the study, and the dipole rejection criteria. While we used an 80% threshold for identifying the somatosensory cortex, higher thresholds may further reduce the distance.

While our algorithm tended to localize to the central and post-central sulcus, there was variability in the localization of the dipoles. This variability could explain the distances observed between localization methods. Additionally, sLORETA has been shown to overestimate the spatial extent of focal sources, and this overestimation may impact the distances observed between methods (Cosandier-Rimélé et al., 2017; Kalina et al., 2023; Hedrich et al., 2017). However, it is encouraging that our distributed source method consistently identified the correct anatomical regions (Fig. 6) with relatively good concordance with the dipole analysis (Fig. 7, Fig. 8). However, our method provides a cortical area instead of a discrete point, resulting in enhanced identification of the somatosensory cortex.

Due to the somatotopic organization of the pre- and post-central gyrus (Sanchez Panchuelo et al., 2018; Meier et al., 2008), our approach in assessing our algorithms’ anatomical accuracy is a bit coarse as we considered the entirety of the pre- and post-central gyrus, as well as the central and post-central sulcus to be correct. However, recent evidence suggests that the somatotopic organization of the pre-central gyrus is interrupted by association cortices, indicating this organization may be more complicated than previously thought (Jensen et al., 2023). Additionally, as these patients are clinically diagnosed with focal or multi-focal epilepsy, there is a possibility of cortical reorganization of eloquent cortices. This phenomenon has not been heavily investigated. Thus, because of this biological variability, we defined source locations that resided anywhere within these cortical regions as anatomically correct when localizing somatosensory responses.

Our algorithm has several unique advantages. In this study, we limited our time of interest for localization to between 15 and 60 ms post-stimulus, as responses in this time range tend to localize to the primary somatosensory cortex. It is worth noting that with median nerve stimulation, responses as late as 250 ms post-stimulus have been observed (Hari and Kaukoranta, 1985). Thus, the first significant advantage of our algorithm is the flexibility to analyze any temporal component of the recorded signal. While we assessed our algorithm by localizing somatosensory cortical areas, a second major advantage is that it can be applied to localize other eloquent areas, such as motor or visual cortices. A third major advantage of our algorithm is the flexibility to analyze data from different recording modalities. This property of our algorithm is particularly appealing as it allows us to use data from a cheaper and more widely used imaging modality compared to MEG, such as high-density EEG, for source localization. While some may interpret the lack of novelty of our algorithm as a weakness, we view this as a strength. We identify the cortical area involved in the task-based response with a heavily validated blind source separation approach, the SVD (Klema and Laub, 1980). The application of SVD offers us two significant advantages. First, SVD reduces the dimensionality of the data by selecting the most dominant mode corresponding to the largest singular value, allowing us to identify the most essential features, in this case, the location and boundary of the activated cortical region. Second, SVD is a model-free factorization approach. Some methods, such as linear regression, attempt to fit data to an underlying model. SVD makes minimal assumptions about the underlying nature of the data, allowing for the identification of the cortical regions whose activation best explains the recorded neuromagnetic response, regardless of the area's boundary, size, or location.

The key innovation of our approach is post-processing the results of a distributed-source inversion method to yield a directly interpretable focal activation region. Using sLORETA or similar techniques without post-processing provides a continuous measure of activation, not a non-binary classification of what tissue is active. Thus, our procedure provides an objective, reproducible procedure for interpreting distributed source results. We selected sLORETA as our distributed-source inversion method based on its generally good performance (Terakawa et al., 2008), with performance similar to that of MNE and dSPM, two other distributed source techniques (Hedrich et al., 2017). While sLORETA, like all source reconstruction methods, has both strengths and weaknesses, it is sufficient for us to accomplish the objective of this manuscript.

We note a few limitations of our study. We analyzed data acquired from patients diagnosed with focal or multi-focal epilepsy, and thus, these patients may have atypical somatosensory representation. Patients receiving MEG for other diagnoses, such as brain tumors, may have a more typical somatosensory representation. Thus, the exact performance for other populations may vary slightly. Another potential limitation in the current study was the time used for dipole fitting. We fit our dipoles at 20 ms and 40 ms post-stimulus, while others in the field will fit dipoles relative to the neuromagnetic response resulting from stimulation (an approximately 5 ms delay). To ensure that our method was not impacted by this choice of dipole localization time, we repeated our analysis to take this 5 ms delay into consideration. We observed no significant differences in dipole localization, dipole parameters, overlap with distributed sources, or distance from distributed sources. While our data was bandpass filtered from 2 Hz to 120 Hz, consistent with how data is processed at our institution, it is also common to filter data from 20 Hz to 250 Hz for the purpose of dipole localization (Antonakakis et al., 2019; Buchner et al., 1994). We repeated our analysis using this alternate filter setting to ensure this choice of bandpass filter did not impact our results. We observed no significant changes in source localization, overlap percentages, or distances to individual dipoles. We did, however, observe a modest increase in the GOF of P20m dipoles with this filter setting. Data for the modified dipole fits and filter parameters are not shown. Another potential limitation is our use of sLORETA as our source reconstruction method. As previously mentioned, sLORETA overestimates the spatial extent of focal sources (Cosandier-Rimélé et al., 2017; Kalina et al., 2023; Hedrich et al., 2017). While other source reconstruction methods, such as coherent maximum entropy on the mean (cMEM), could have been used, this method has shown a similar distance from the epileptic focus compared to sLORETA (Pellegrino et al., 2018). Future work could examine the differences in source localization between sLORETA and cMEM in a cohort of epilepsy patients. Beamforming approaches such as 4-ExSo-MUSIC could also be used. However, beamforming approaches are better for deeper sources with large signal-to-noise ratios (Chowdhury et al., 2016). While other methods, such as FAST-IRES exist, the method has been applied to scalp EEG for spike localization (Sohrabpour et al., 2020). This method has not yet been modified to operate on MEG data, and thus, a direct comparison between methods could not be performed. A final limitation of our approach is that our study is retrospective. To further validate our method of somatosensory localization, our noninvasive approach should be compared to cortical stimulation mapping, the clinical gold standard for the localization of eloquent cortices. However, this comparison was not possible due to the limited data available for the retrospective patients. Future studies could compare the localization of eloquent cortices between invasive and noninvasive approaches.

Our algorithm successfully localized the somatosensory cortex and provides a defined cortical area, in contrast to the single point identified by ECD analysis. Having thus demonstrated the utility of our algorithm in identifying the somatosensory cortex, we conclude that our algorithm is ready for testing using other task-based MEG recordings. Looking to the future, our results open the door for research comparing our results with cortical stimulation mapping, a clinical gold standard. Comparing the algorithm to cortical stimulation can be used to assess its sensitivity and specificity on a global scale and further validate MEG as a viable tool for non-invasive localization of eloquent cortices. Algorithm localization can be used, for example, to provide a location for cortical stimulation, either invasively, with stimulating electrodes or non-invasively, with transcranial magnetic stimulation, to elicit a desired physiological or behavioral response. Our results thus open the possibility for research into automated, non-invasive methods for identifying a broad range of eloquent cortices, adding essential information for presurgical planning, which could lead to better postoperative outcomes for patients undergoing surgical intervention to treat various neurological disorders. While we have focused on clinical applications, our method would also provide an objective, distributed source measure that could be used for multiple research areas. For example, our algorithm could be used to examine the localization differences between healthy controls and clinical populations to investigate the effect of diseases or to compare source localization to the cortical homunculus to examine individual differences in cortical wiring.

## Compliance with ethical standards

This study was performed in a manner consistent with the United States Federal Policy for the Protection of Human Subjects (the Common Rule). Approval was granted by the local Institutional Review Board (IRB) of the University of Nebraska Medical Center in November 2021 (protocol #0714-21-EP). Consistent with local and federal policy, the IRB determined that participant consent for this retrospective study was not required.

## CRediT authorship contribution statement

**Kevin Tyner:** Writing – review & editing, Writing – original draft, Formal analysis, Conceptualization. **Matthew McCumber:** Writing – review & editing, Formal analysis. **Srijita Das:** Writing – review & editing, Formal analysis. **Carmen Urban:** Writing – original draft, Formal analysis. **Anthony J. Maxin:** Writing – review & editing, Formal analysis. **Tiffany Chu:** Writing – review & editing, Formal analysis. **Mustaffa Alfatlawi:** Writing – review & editing, Formal analysis, Conceptualization. **Stephen V. Gliske:** Writing – review & editing, Supervision, Data curation, Conceptualization.

## Declaration of competing interest

Anthony J. Maxin would like to disclose equity interest in Apertur, Inc. The rest of the authors have no relevant financial disclosures.

## Data Availability

The authors do not have permission to share data.
